# Healthy Aging Men Do Not Suffer From Relevant Limitations of Their Reproductive Functions

**DOI:** 10.1111/andr.70147

**Published:** 2025-11-07

**Authors:** Simone Bier, Jann‐Frederik Cremers, Phillip Schrage, Amelie Körtje, Claudia Krallmann, Sabine Kliesch, Jörg Gromoll, Sandra Laurentino, Michael Zitzmann

**Affiliations:** ^1^ Department of Andrology, Centre of Reproductive Medicine and Andrology University Clinic and University of Münster Münster Germany

**Keywords:** erectile function, hypogonadism, male reproductive aging, metabolic health, semen parameters, testosterone decline

## Abstract

**Background:**

While metabolic disorders are well‐established contributors to testosterone decline and erectile dysfunction (ED), little is known about the natural progression of reproductive parameters in healthy aging men.

**Objectives:**

This study aimed to evaluate longitudinal changes in reproductive parameters and sought to determine the influence of body mass index (BMI) and glycated hemoglobin (HbA1c) on these changes.

**Patients and Methods:**

A total of 197 healthy men (aged 18–84 years) were assessed at baseline (FAMe 1), with 117 participants returning for follow‐up (FAMe 2) after approximately 6 years. All participants underwent a thorough andrological examination.

**Results:**

Total and free testosterone levels declined significantly over 6 years in men older than 25 years at baseline (*p* < 0.05). FSH levels increased significantly in men older than 35 years at FAMe 1 (*p* < 0.05), while LH and SHBG remained unchanged. Despite moderate declines, semen parameters remained mostly within physiological limits. Erectile function exhibited a moderate but progressive decline (*p* < 0.001). In men over 45 years, neither age (*p* = 0.13) nor testosterone (*p* = 0.52) influenced erectile function, while an increment of HbA1c levels was significantly associated with deteriorating erectile function (*p* = 0.002). Similarly, AMS scores significantly increased after age 45, which was strongly correlated with higher HbA1c levels (*p* = 0.001).

**Discussion:**

While testosterone levels declined with aging, they remained within the normal range, suggesting that healthy men experience only apparently mild reproductive aging. Erectile function and hypogonadism‐like symptoms were more strongly associated with glycemic control than with testosterone levels.

**Conclusion:**

In healthy men, reproductive and sexual aging occur gradually, with testosterone and semen parameters largely preserved over time. However, metabolic health, rather than testosterone, plays a key role in the progression of ED and hypogonadism‐like symptoms, emphasizing the need for preventive metabolic interventions to maintain reproductive health.

## 1 Introduction

Since the mid‐20th century, rising life expectancy in industrialized societies has intensified interest in aging with optimal health and quality of life [1]. Among the most frequently reported concerns of aging men is hypogonadism, particularly functional hypogonadism, a condition characterized by symptoms such as diminished libido and erectile dysfunction (ED), in conjunction with low serum testosterone levels [1, 2, 3, 4, 5]. Increasingly, the interplay between hypogonadism, ED, obesity, and diabetes mellitus has drawn attention, given its well‐established links to cardiovascular disease, diminished quality of life, and long‐term health consequences [6].

In 2013, Corona et al. demonstrated that obesity (BMI > 30 kg/m^2^) triples the risk of functional hypogonadism. Furthermore, obesity in combination with type 2 diabetes mellitus (T2DM) exacerbates suppression of the hypothalamic–pituitary–testicular axis, amplifying testosterone deficiency [7]. The association between ED (as assessed by the IIEF score) and HbA1c levels in T2DM patients is well documented [8]. Notably, metabolic dysfunction exerts a greater influence on the risk of hypogonadism and its related symptoms than age‐related testicular senescence [9]. Additionally, it has been shown that subfertile men have a significantly increased risk of hypogonadism compared with healthy men. This hypogonadism is associated with elevated HbA1c levels and reduced bone density [10]. However, longitudinal data examining the trajectory of erectile function and hypogonadism‐related symptoms in healthy men, as well as their correlation with total and free testosterone levels over time, remains absent.

Age‐related fertility decline in men has become an increasingly relevant topic, particularly given the societal trend toward delayed parenthood. Studies examining the effects of aging on semen parameters have reported reductions in ejaculate volume, sperm concentration, total sperm count, motility, and normal sperm morphology [11, 12]. However, most of these studies derive from infertility clinic datasets, limiting their generalizability to healthy men.

In 2020, our group published the first cross‐sectional analysis of semen parameters and hormonal profiles in a cohort selected for optimal general health—‐the FAMe (Fertility in Aging Healthy Men) cohort [13]. Our findings revealed that, while spermatozoa from older men exhibited epigenetic alterations and increased DNA damage, traditional semen parameters and hormonal levels remained within physiological ranges. However, longitudinal data tracking the evolution of these parameters in healthy men over time was still lacking.

In this study, we sought to fill this gap by investigating longitudinal changes in clinical, hormonal, and ejaculate parameters over a 6‐year period in the healthy men of the FAMe cohort.

## 2 Materials and Methods

### 2.1 Ethics

The study was approved by the Ethics Committee of the Medical Faculty, University of Münster (approval number: 2013‐255‐f‐S). All participants provided written informed consent prior to inclusion in the study.

### 2.2 Cohort Recruitment and Baseline Assessment

The FAMe (Fertility in Aging Healthy Men) cohort was initially recruited between October 2014 and April 2016 through public advertisement, followed by a structured preselection process involving questionnaires and clinical evaluations. We wanted to include 200 male participants, aged between 18 and 80 years without relevant pre‐existing health conditions. Prospective participants were required to complete detailed health questionnaires, allowing for the exclusion of individuals with any of the following conditions: smoking within the previous year, illegal drug use, regular medication use (exceptions: for mild hypertension, hypothyroidism, and dyslipoproteinemia), hospitalization within the previous month, current or previous cancer, or history of cancer treatment, severe chronic renal failure, chronic viral infections, congenital malformations or previous urogenital surgeries, prior diagnosis or treatment for fertility impairment, chromosomal abnormalities, participation in clinical trials within the year prior. Given that nearly all participants over the age of 50 who presented for enrolment in our study were receiving treatment with at least one statin and/or an antihypertensive agent, we resolved to admit individuals with arterial hypertension provided it was adequately controlled by a single antihypertensive medication, as well as those undergoing prophylactic statin therapy. Moreover, in view of the fact that approximately 5% of the German population is affected by hypothyroidism, we elected to include individuals with well‐controlled hypothyroidism in the study cohort [14].

All participants underwent a detailed anthropometric, endocrine, and andrological assessment, alongside questionnaire‐based evaluations addressing their well‐being and sexual health. This initial time point is referred to from here onward as FAMe 1.

### 2.3 General Examination

To assess general physical health, height and weight were measured at both time points, and BMI was calculated accordingly. All participants underwent a comprehensive evaluation, which included a detailed medical history (anamnesis). Erectile function, loss of libido, and ejaculatory disorders were assessed by using validated questionnaires as International Index of Erectile Function‐Erectile Function (IIEF‐EF) as well as Aging‐Male‐Symptom Score (AMS).

### 2.4 Assessment of the Gonadotropins and Sex Steroids

The venous blood sample was drawn by the examining physician. Post‐collection, serum was promptly separated via centrifugation at a force of 800 × *g*, followed by immediate snap‐freezing and storage at a temperature of −20°C to preserve sample integrity. Serum values of FSH, LH, testosterone, SHBG, estradiol and prolactin were measured on an Abbot Architect i1000SR automated analyzer (Abbott Diagnostics) with the chemiluminescent microparticle immunoassay. Quantitative analysis of serum testosterone levels was conducted employing a commercially available enzyme‐linked immunosorbent assay (ELISA) kit, procured from DRG Instruments GmbH, Marburg, Germany. This specific immunoassay for testosterone undergoes routine calibration on a quarterly basis, aligned against reference standards established through liquid chromatography–mass spectroscopy (LCMS–MS). The assay consistently surpasses quality control benchmarks, demonstrating the ability to replicate the outcomes yielded by mass spectroscopy. It exhibits an imprecision margin of less than 10% within the serum testosterone concentration range of 5–20 nmol/L.

Furthermore, the assay's reliability is underscored by its high reproducibility, evidenced by intra‐assay coefficients of variation (CVs) that are less than 2%, and mean inter‐assay CVs maintained below 5%. This level of precision and consistency in measurement ensures the robustness and validity of the testosterone level assessments conducted in this study.

The determination of free testosterone levels was conducted through a calculative approach, leveraging the concentrations of sex hormone‐binding globulin (SHBG) and total serum testosterone. This calculation adhered to the method delineated by Vermeulen, a recognized standard in the field.

In addition to testosterone, serum concentrations of SHBG, luteinizing hormone (LH), follicle‐stimulating hormone (FSH), and prostate‐specific antigen (PSA) were assayed utilizing highly specific time‐resolved fluoro‐immunoassays. These assays were performed with equipment sourced from Autodelfia, Freiburg, Germany.

The robustness and reproducibility of these methods are evidenced by their analytical performance: mean intra‐assay CVs were maintained below 2%, and mean inter‐assay CVs were kept under 5%. Furthermore, to ensure continued accuracy and reliability, all assays are subjected to stringent external quality control measures on a quarterly basis. These quality assessments are conducted in a blinded manner, meaning the laboratory analyzing the quality control samples is not aware of the expected results. This practice is crucial for unbiased evaluation. The assays regularly meet and surpass these external quality control standards. The HbA1c value was measured at the Central Laboratory of the University hospital.

### 2.5 Semen Analysis

For semen analysis and reasons of consistency, the fifth edition of the WHO guidelines was used, because sixth edition had not appeared yet when the first examination was performed [15]. Briefly, the sperm concentration was measured in duplicates of 10 µL each using a hemocytometer. Motility assessment involved placing two 10 µL samples of native ejaculate under a 22 mm × 22 mm coverslip and 200 spermatozoa each were counted per sample. Motile spermatozoa were categorized into: (1) progressive motile (PR), which includes fast progressive (a) and slow progressive (b), (2) non‐progressive (NPR), and immotile spermatozoa (IM). Vitality was assessed using eosin red staining, and sperm morphology was evaluated using Papanicolaou staining to establish baseline values. For further evaluations of the semen parameters the Reference values of the sixth edition of the WHO manual were used [16].

### 2.6 Longitudinal Follow‐Up

To obtain longitudinal data, all previously enrolled participants were re‐contacted in 2020 via postal invitation and invited for a follow‐up assessment. This second time point is referred to as FAMe 2.

For comparative analyses, participants were stratified into six age groups based on their age at FAMe 1: Group 1, 18–25 years; Group 2, 26–35 years; Group 3, 36–45 years; Group 4, 46–55 years; Group 5: 56–65 years; Group 6: > 65 years (Table 1).

**TABLE 1:** Number and age of participants at first evaluation per group.

Group	Age at FAMe 1 (years)	Number of participants (FAMe 1)	Number of participants (FAMe 2)
1	18–25	34	12
2	26–35	36	18
3	36–45	28	20
4	46–55	39	27
5	56–65	37	27
6	> 65	23	13

Abbreviations: FAMe 1, first evaluation period; FAMe 2, second evaluation period.

We only include the data of the participants who introduced themselves for the examination of FAMe 1.0 as well as Fame 2.0.

### 2.7 Statistical Analysis

All statistical analyses were conducted using SPSS (version 27.0, IBM, Armonk, NY, USA). Prior to applying parametric tests, the distribution of continuous variables was evaluated using Shapiro–Wilk and Kolmogorov–Smirnov tests. Skewness and kurtosis were assessed to determine deviations from normality. In cases of significant skewness and heteroscedasticity, logarithmic transformations were performed to normalize the data distribution. If transformation failed to correct the distribution, appropriate non‐parametric tests were used instead. We used *t*‐tests to compare patient and semen characteristics over time. For the analysis of standardized questionnaire data, we used the Wilcoxon–Mann–Whitney test.

To evaluate influencing factors on outcomes, we employed linear mixed models (LMMs), as their use was necessitated by the dependence of residuals. Random effect variables included the intercept and patient identity, allowing for the consideration of intra‐individual variability across time points. The assumption of homoscedasticity was examined using Levene's test, and variance inflation factors (VIFs) were assessed to detect potential collinearity among predictors.

## 3 Results

Following the preselection process, 265 eligible volunteers were invited to the outpatient clinic for a comprehensive medical evaluation and biological sample collection. Of these, 200 individuals agreed to participate, and ultimately, 197 men (aged 18–84 years) were included in the study FAMe 1.0.

**FIGURE 1:**
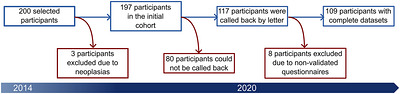
Recruitment of the participants.

For the longitudinal setting 117 individuals (group 1: *n* = 12, group 2: *n* = 18, group 3: *n* = 20, group 4: *n* = 27, group 5: *n* = 27, group 6: *n* = 13) agreed to participate and underwent the same comprehensive clinical and biological evaluations as in FAMe 1 (Figure 1). Thus, 83 men were lost to follow‐up. Of the 117 re‐visiting men, 109 participants completed all questionnaires. The semen parameters for one of the participants were excluded, as he had undergone a vasectomy between the two assessment periods.

The rationale for our age stratification was grounded in the clinical realities of male reproductive health. Infertility typically manifests in men younger than 40 years, whereas ED more commonly emerges beyond this age. By delineating groups along this threshold, we were able to capture these distinct yet complementary facets of male reproductive function. At the same time, our objective was not merely to contrast these age brackets, but to encompass a broader spectrum of male reproductive health and to evaluate trajectories across different life stages. Importantly, the chosen group sizes also afforded us sufficient flexibility to merge cohorts for more granular subgroup analyses, as demonstrated in our regression models.

### 3.1 General Health

Across most age groups, BMI remained stable over time. However, we observed a significant increase in BMI in Group 4 at FAMe 2 compared with FAMe 1 (*p* = 0.05), whereas no significant changes were detected in the other age groups.

### 3.2 Glycated Hemoglobin

Glycated hemoglobin (HbA1c) is a clinical marker of cumulative glycemic exposure and is instrumental in predicting long‐term metabolic complications. Over the course of the study, we observed a significant decrease in HbA1c in Group 2 (*p* < 0.001). Despite this decline, HbA1c levels at both FAMe 1 and FAMe 2 remained within the normal physiological range, irrespective of participant age.

### 3.3 Gonadotropins and Sex Steroids

To assess hormonal variations over time in healthy men, serum concentrations of gonadotropins and sex steroids were analyzed at FAMe 1 and FAMe 2. LH levels remained within the normal physiological range across all age groups and exhibited no significant changes over time (Table 2). In contrast, FSH concentrations showed a significant increase in participants older than 35 years at baseline. This increase was statistically significant in Group 3 (*p* = 0.006), Group 4 (*p* = 0.002), Group 5 (*p* = 0.013), and Group 6 (*p* = 0.01). However, in the two youngest groups (younger than 35 years at enrollment), FSH levels remained stable. Despite this age‐related increase, FSH concentrations remained within the normal range in all participants, including those in the older age groups (Table 3).

**TABLE 2:** Serum luteinizing hormone (LH; reference range 2–10 U/L) of the participants at the two evaluation periods.

	FAMe 1		FAMe 2		
Group	Mean LH (U/L)	SD	Mean LH (U/L)	SD	*p* value
1 (*n* = 12)	3.06	0.88	3.00	1.23	0.83
2 (*n* = 18)	2.94	1.13	2.78	1.21	0.45
3 (*n* = 20)	3.38	1.44	3.36	1.25	0.97
4 (*n* = 27)	3.07	1.13	3.07	1.38	0.97
5 (*n* = 27)	2.60	1.07	2.83	1.16	0.21
6 (*n* = 13)	3.59	1.35	3.27	1.20	0.29

FAMe 1, first evaluation period; FAMe 2, second evaluation period.

**TABLE 3:** Serum follicle‐stimulating hormone (FSH; reference range 1–7 U/L) of the participants at the two evaluation periods.

	FAMe 1		FAMe 2		
Group	Mean	SD	Mean	SD	*p* value
1 (*n* = 12)	3.33	2.14	3.50	3.03	0.71
2 (*n* = 18)	3.54	1.25	3.82	1.28	0.08
3 (*n* = 20)	3.91	1.95	4.61	2.18	**0.006**
4 (*n* = 27)	4.19	2.70	4.67	2.88	**0.002**
5 (*n* = 27)	4.22	2.24	5.80	4.50	**0.013**
6 (*n* = 13)	5.24	1.62	6.17	2.36	**0.01**

*p* values < 0.05 were considered significant.

Total testosterone levels showed significant changes in all participants older than 25 years at FAMe 1, with significant declines in Group 2 (*p* = 0.002), Group 3 (*p* = 0.014), Group 5 (*p* = 0.02), and Group 6 (*p* = 0.01). However, despite these changes, total testosterone levels remained within the normal range, independent of age or time point of measurement (Table 4). Consistent with this, free testosterone levels also remained within physiological limits throughout the study. However, significant declines were observed in Group 2 (*p* = 0.02) and Group 3 (*p* = 0.012; Table 5). Although dihydrotestosterone (DHT) levels were within the normal range in all groups at both FAMe 1 and FAMe 2, we detected a significant decrease across all age groups (Table 6).

**TABLE 4:** Total testosterone (reference range > 12 nmol/L) of the participants at the two evaluation periods.

	FAMe		FAMe2		
Group	Mean	SD	Mean	SD	*p* value
1 (*n* = 12)	23.63	9.09	20.24	8.56	0.13
2 (*n* = 18)	23.74	5.83	19.08	5.55	**0.002**
3 (*n* = 20)	22.76	6.81	18.89	6.31	**0.014**
4 (*n* = 27)	19.36	5.97	18.34	5.12	0.169
5 (*n* = 27)	20.80	5.40	19.28	5.48	**0.02**
6 (*n* = 13)	24.39	8.65	20.45	7.17	**0.01**

*p* values < 0.05 were considered significant.

**TABLE 5:** Free testosterone (reference range > 250 pmol/L) of the participants at the two evaluation periods.

	FAMe 1		FAMe 2		
Group	Mean	SD	Mean	SD	*p* value
1 (*n* = 12)	417.08	142.43	386.00	164.68	0.054
2 (*n* = 18)	493.06	123.08	405.77	97.82	**0.002**
3 (*n* = 20)	460.65	129.38	377.60	88.14	**0.012**
4 (*n* = 27)	392.56	93.27	385.81	114.02	0.76
5 (*n* = 27)	337.04	78.27	337.07	101.52	0.99
6 (*n* = 13)	421.08	129.43	347.23	80.18	0.10

*p* values < 0.05 were considered significant.

**TABLE 6:** Dihydrotestosterone (reference range > 0.5 nmol/L) of the participants at the two evaluation periods.

	FAMe 1		FAMe 2		
Group	Mean	SD	Mean	SD	*p* value
1 (*n* = 12)	0.86	0.26	0.58	0.24	**0.009**
2 (*n* = 18)	0.88	0.30	0.60	0.16	**< 0.001**
3 (*n* = 20)	0.76	0.22	0.62	0.21	**< 0.045**
4 (*n* = 27)	0.86	0.34	0.58	0.21	**< 0.001**
5 (*n* = 27)	0.80	0.28	0.61	0.30	**0.008**
6 (*n* = 13)	0.95	0.35	0.62	0.20	**0.003**

*p* values < 0.05 were considered significant.

SHBG levels remained unchanged across all groups and within normal limits throughout the evaluated period (Table 7).

**TABLE 7:** Sex hormone‐binding globulin (SHBG; reference range 11–71 nmol/L) of the participants at the two evaluation periods.

	FAMe 1		FAMe 2		
Group	Mean	SD	Mean	SD	*p* value
1 (*n* = 12)	38.75	15.49	36.00	15.73	0.21
2 (*n* = 18)	35.89	12.41	34.17	12.21	0.58
3 (*n* = 20)	37.55	12.15	38.05	12.42	0.75
4 (*n* = 27)	44.78	16.95	41.33	17.09	0.12
5 (*n* = 27)	45.70	15.54	44.41	16.03	0.44
6 (*n* = 13)	50.00	18.27	43.00	14.46	0.10

### 3.4 Semen Parameters

Ejaculate volume remained within the normal range across all evaluated age groups. However, a significant and progressive decline in ejaculate volume was observed over time in all age groups, with the exception of the oldest group (≥ 65 years at FAMe 1), where no significant change was detected (Table 8).

**TABLE 8:** Semen analysis—Ejaculate volume (reference range > 1.4 mL) of the participants at the two evaluation periods [16].

	FAMe 1		FAMe 2		
Group	Mean	SD	Mean	SD	*p* value
1 (*n* = 12)	4.38	1.36	3.51	1.40	**0.047**
2 (*n* = 18)	4.82	2.38	3.99	2.47	**0.007**
3 (*n* = 20)	3.55	1.27	2.98	1.42	**0.016**
4 (*n* = 27)	3.39	1.44	2.37	1.11	**< 0.001**
5 (*n* = 27)	2.98	1.38	2.23	1.21	**< 0.001**
6 (*n* = 12)	2.00	0.91	1.72	0.71	0.113

*p* values < 0.05 were considered significant.

Sperm concentration remained stable over time, with no significant decline observed in any age group. Both mean total sperm count and mean sperm concentration remained within physiological levels across all age groups at both FAMe 1 and FAMe 2 timepoints. Nevertheless, a statistically significant decrease in total sperm count was identified in Group 3 (*p* = 0.001) and Group 4 (*p* = 0.005) over the study period (Tables 9 and 10).

**TABLE 9:** Semen analysis—Total sperm count (reference range ≥ 39 Mio) of the participants at the two evaluation periods [16].

	FAMe 1		FAMe 2		
Group	Mean	SD	Mean	SD	*p* value
1 (*n* = 12)	181.42	123.16	144.29	102.93	0.20
2 (*n* = 18)	175.98	115.57	164.02	133.11	0.75
3 (*n* = 20)	117.94	67.38	73.02	60.61	**0.001**
4 (*n* = 27)	161.83	141.65	110.43	105.10	**0.005**
5 (*n* = 27)	121.36	114.88	94.24	120.73	0.242
6 (*n* = 12)	186.90	130.39	123.20	132.50	0.075

*p* values < 0.05 were considered significant.

**TABLE 10:** Semen analysis—Sperm concentration (reference range ≥ 16 Mio/mL) of the participants at the two evaluation periods [16].

	FAMe 1		FAMe 2		
Group	Mean	SD	Mean	SD	*p* value
1 (*n* = 12)	45.22	35.83	42.73	29.53	0.73
2 (*n* = 18)	39.27	25.70	41.33	35.36	0.82
3 (*n* = 20)	35.55	24.91	26.98	24.96	0.10
4 (*n* = 27)	47.41	39.73	45.07	23.83	0.68
5 (*n* = 27)	46.01	40.30	43.03	45.58	0.67
6 (*n* = 12)	85.10	51.80	66.50	67.53	0.25

Progressive (a + b) sperm motility declined significantly in all age groups between FAMe 1 and FAMe 2. However, mean motility values fell below the normal range at FAMe 2 only in the two oldest age groups (Group 5 and Group 6; Table 11).

**TABLE 11:** Semen analysis—A + B‐Motility (reference range ≥ 30%) of the participants at the two evaluation periods [16].

	FAMe 1		FAMe 2		
Group	Mean	SD	Mean	SD	*p* value
1 (*n* = 12)	54.96	12.78	46.79	9.50	**0.003**
2 (*n* = 18)	55.78	6.58	43.47	14.61	**0.003**
3 (*n* = 20)	48.63	11.72	39.33	19.18	**0.031**
4 (*n* = 27)	49.61	12.40	38.32	16.93	**< 0.001**
5 (*n* = 27)	44.67	13.80	30.44	16.87	**< 0.001**
6 (*n* = 12)	40.33	17.41	31.63	16.97	**0.004**

*p* values < 0.05 were considered significant.

Sperm vitality remained within normal limits in most age groups with the exception of Group 6 at FAMe 1, where a notably lower sperm vitality was recorded. Interestingly, this decline was not present at FAMe 2. Furthermore, sperm vitality significantly improved over time in Group 3 (*p* < 0.05) and Group 6 (*p* < 0.05; Table 12).

**TABLE 12:** Semen analysis—Vitality (reference range ≥ 54%) of the participants at the two evaluation periods [16].

	FAMe 1		FAMe 2		
Group	Mean	SD	Mean	SD	*p* value
1 (*n* = 11)	73.55	10.60	78.00	6.08	0.24
2 (*n* = 17)	73.44	4.10	75.18	6.70	0.36
3 (*n* = 17)	66.56	9.57	73.53	6.43	**0.005**
4 (*n* = 24)	67.10	9.23	70.10	7.07	0.151
5 (*n* = 25)	62.62	11.80	65.48	12.50	0.24
6 (*n* = 12)	55.50	19.61	61.13	17.29	**0.043**

*p* values < 0.05 were considered significant.

### 3.5 International Index of Erectile Function

**FIGURE 2:**
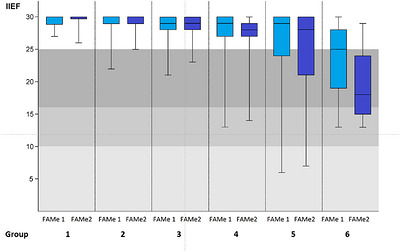
Change of the IIEF‐score between the different groups. IIEF‐Score 26–30: no erectile dysfunction (ED), 22–25: mild ED, 17–21: mild to moderate ED, 11–16: moderate ED, and 6–10: severe ED.

The IIEF‐EF score across all participants was 27.2 ± 4.6 at FAMe 1 and 26.4 ± 5.3 at FAMe 2. No statistically significant changes were observed over time within any age group (Group 1: *p* = 0.85; Group 2: *p* = 0.76; Group 3: *p* = 0.94; Group 4: *p* = 0.70; Group 5: *p* = 0.59; Group 6: *p* = 0.17). Moreover, none of the participants exhibited severe ED at either time point and moderate ED was diagnosed exclusively in participants from the three oldest age groups (≥ 45 years at FAMe 1; Groups 4, 5, and 6; Figure 2).

### 3.6 Aging‐Male‐Symptom Score

**FIGURE 3:**
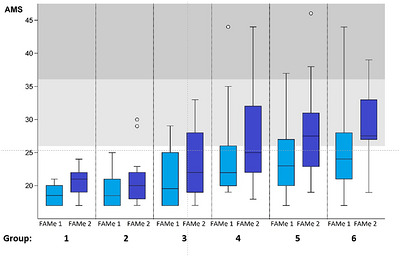
Change of Aging‐Male Symptoms between the different groups. AMS Score 17–26: no symptoms, 27–36: mild symptoms, 37–49 moderate symptoms, and > 50 severe symptoms (> 50).

To evaluate hypogonadism‐related symptoms, participants completed AMS questionnaire at both time points. The mean AMS score was 22.3 ± 5.4 at FAMe 1 and 25.3 ± 6.7 at FAMe 2. A statistically significant worsening of AMS scores was observed in Group 5 (*p* = 0.04), indicating an increased perception of aging‐related symptoms over time in this age group. However, most participants across all age groups reported either no or only mild symptoms, irrespective of age or time point (Figure 3).

### 3.7 Additional Factors Influencing Erectile Function

To assess whether BMI, HbA1c, or serum concentrations of sex steroids were associated with age‐related decline in erectile function, we applied mixed linear models (LMMs). We observed a significant association between age group and erectile function, with participants in Groups 4 (*p* = 0.01), 5 (*p* < 0.001), and 6 (*p* < 0.001) showing a progressive decline in erectile function over time. However, total testosterone levels (*p* = 0.13) and HbA1c (*p* = 0.11) did not exhibit a significant influence on the deterioration of erectile function with aging.

To further isolate factors influencing erectile function in aging men, we performed an additional analyses exclusively in participants who were ≥ 45 years old at FAMe 1 (Groups 4, 5, and 6). Within this older age subgroup, neither age (*p* = 0.13) nor total testosterone levels (*p* = 0.52) had a statistically significant impact on erectile function. However, HbA1c levels were significantly associated with IIEF scores (*p* = 0.002), suggesting that glycemic control may play a more crucial role in erectile function in aging men than either age or testosterone levels.

### 3.8 Factors Influencing Aging Male Symptoms

To determine whether BMI, HbA1c, or serum concentrations of sex steroids were associated with longitudinal changes in AMS scores, we applied mixed linear models (LMMs). No significant associations were observed between total testosterone (*p* = 0.45), HbA1c (*p* = 0.99), or younger age groups (< 35 years; Group 1: *p* = 0.81, Group 2: *p* = 0.13) and AMS scores over time. However, we observed a significant worsening of AMS scores in participants older than 45 years (*p* < 0.001), suggesting that aging beyond this threshold is a critical factor for the development of symptoms usually attributed to hypogonadism.

To further explore this effect, we performed a separate analysis of participants aged ≥ 45 years at FAMe 1 (Groups 4, 5, and 6). Within this older age subset, AMS scores worsened significantly over time and were strongly associated with HbA1c levels (*p* = 0.001). Notably, this effect was independent of chronological age (*p* = 0.57) and total testosterone levels (*p* = 0.18), indicating that glycemic status may play a more significant role than testosterone in the progression of aging‐related symptoms in men over 45 years and that older men might exhibit hypogonadism‐like symptoms in the absence of bona fide hypogonadism.

## 4 Discussion

With increasing life expectancy and the growing trend of delayed parenthood, the preservation of reproductive health and quality of life has gained significant importance [17]. Despite this, relatively little is known about the natural progression of reproductive health in aging, yet healthy, men.

Our longitudinal study provides valuable insights into this topic. We demonstrate that serum concentrations of sex steroids and semen parameters remain within normal ranges, independent of age. Furthermore, the quality of life of our participants, even among the oldest age groups, was not negatively impacted by severe ED or pronounced hypogonadism‐like symptoms. However, we observed a significant decline in testosterone levels over the 6‐year follow‐up period in all participants who were older than 25 years at FAMe 1, indicating that male reproductive aging begins relatively early in adulthood. Despite this decline, testosterone concentrations remained within the normal range across all age groups, suggesting that this process is gradual rather than abrupt.

Previous studies have reported that older men often exhibit lower testosterone levels, primarily attributed to testicular aging and reduced Leydig cell function [9, 18]. However, emerging evidence suggests that metabolic disorders, such as diabetes and obesity, exert a more profound impact on testosterone levels than aging itself [9]. Our findings reinforce this perspective, as testosterone levels remained within physiological ranges, even in older age groups, highlighting the importance of metabolic rather than chronological aging.

Interestingly, while some studies have reported an age‐related increase in SHBG, particularly in men over 45 years [12, 19], we could not observe any detectable rise in SHBG in the FAMe cohort, even among older participants. This supports the hypothesis that elevated SHBG levels are not an inevitable consequence of aging itself, but may instead be driven by age‐associated metabolic dysfunctions, such as increased adiposity [12, 20].

Advanced paternal age has been linked to lower fertility rates, higher miscarriage risks, and increased offspring health complications [21]. Several studies have reported that ejaculate volume and sperm concentration decline with advancing age. However, in FAMe 1 [13], no significant age‐related differences in sperm parameters were observed. This finding was confirmed in our longitudinal analysis, which showed that ejaculate volume, total sperm count, and sperm concentration remained within normal ranges across all age groups.

While routine semen parameters were largely preserved, sperm motility declined significantly over time, consistent with prior finding [12, 22, 23]. However, only participants in Groups 5 and 6 (≥ 56 years at FAMe 1) exhibited motility values below the normal range. This reinforces the hypothesis that, while semen parameters decline with age, their clinical impact on fertility is likely limited in metabolically healthy men.

The impact of aging on spermatogenesis remains controversial, as it is difficult to distinguish the effects of aging‐associated morbidities from aging itself [24].

ED is a well‐established marker of vascular and metabolic health, often associated with cardiovascular disease, reduced quality of life, and psychological distress [12, 25, 26, 27]. Interestingly, in our cohort, no severe ED was diagnosed at any age, although we observed a moderate and progressive decline in erectile function over time, consistent with previous studies [28].

A key finding of our study is that, in this cohort of healthy men, age and testosterone levels are not significantly associated with erectile function in men over 45 years. Instead, HbA1c levels were strongly correlated with erectile function, despite being within the normal range. This aligns with prior evidence suggesting that ED can serve as an early marker of glycemic disturbances, including prediabetes and diabetes mellitus [29]. The association between HbA1c and ED is well documented, and our findings suggest that even in metabolically healthy men, subtle variations in glycemic control may influence erectile function.

Furthermore, testosterone levels did not significantly affect erectile function in either the full cohort or in the subgroup of men aged ≥ 45 years at FAMe 1, reinforcing the notion that metabolic health exerts a stronger influence on erectile function than testosterone levels alone in otherwise healthy men. While hypogonadism and ED are closely linked in men with diabetes [30], our results suggest that further research is needed to elucidate the underlying mechanisms driving this association in otherwise healthy men.

To accurately define hypogonadism, specific hypogonadism‐related symptoms must be present, rather than relying solely on testosterone levels [5, 6, 31]. The most clinically relevant symptoms include a decrement of libido and impaired erections, particularly when testosterone values fall below normal thresholds [32]. Therefore, in addition to erectile function, we assessed aging male symptoms using the AMS (Aging Male Symptom) questionnaire.

The AMS score increased significantly with age, reflecting a greater burden of aging‐related symptoms. However, when we examined only participants ≥ 45 years at FAMe 1, neither age nor testosterone levels influenced AMS scores. Instead, HbA1c levels were significantly associated with worsening AMS scores, suggesting a metabolic rather than endocrine driver of aging symptoms.

Although the precise pathophysiological mechanisms remain unclear, it is well established that T2DM is associated with reduced testosterone levels, ED, depressive symptoms, and decreased libido [30]. Our findings suggest that even in otherwise healthy men, metabolic factors influence endocrine function and subjective well‐being.

Metabolic health is determined by an ensemble of parameters, including glycemic control, lipid profile, blood pressure, waist circumference, and insulin sensitivity. Within our selected cohort, we observed that changes of HbA1c emerged as a significant predictor of both deterioration in the AMS score and decline in erectile function among men older than 45 years. Notably, these HbA1c values remained within the conventional reference range. We therefore posit that men exhibiting such relative shifts in HbA1c may already harbor subclinical impairments in insulin sensitivity and, consequently, carry an elevated long‐term risk for the development of metabolic syndrome.

## 5 Conclusion

Our findings indicate that even subtle elevations in glycemic markers within the normoglycemic range are associated with a measurable decline in erectile function. This observation suggests that glycemic indices—particularly HbA1c—may serve as early biomarkers for incipient vascular or neurogenic changes that precede clinically manifest ED. Accordingly, the progressive deterioration of erectile performance might reflect a heightened sensitivity of penile endothelial and neural structures to glycemic fluctuations, even before overt hyperglycemia is present. These results underscore the potential utility of glycemic parameters as early indicators of impending sexual health disturbances and broader cardiometabolic sequelae.

## Funding

This research was funded by the German Federal Ministry for Education and Research (BMBF) as part of the project ReproTrack.MS—Centre for Research and Development of Reproductive Scientists (GRANT Number: 01GR2303).

## Conflict of Interest

None of the authors have a conflict of interest to disclose.
